# Causes of Childhood Cancer: A Review of the Recent Literature: Part I—Childhood Factors

**DOI:** 10.3390/cancers16071297

**Published:** 2024-03-27

**Authors:** Angela M. Ricci, Rebecca T. Emeny, Pamela J. Bagley, Heather B. Blunt, Mary E. Butow, Alexandra Morgan, Jennifer A. Alford-Teaster, Linda Titus, Raymond R. Walston, Judy R. Rees

**Affiliations:** 1 Department of Pediatrics, Division of Pediatric Hematology-Oncology, Dartmouth Health Childrens, Lebanon, NH 03756, USA; 2 Department of Internal Medicine, Division of Molecular Medicine, UNM Comprehensive Cancer Center, Cancer Control & Population Sciences Research Program, University of New Mexico Health Sciences, Albuquerque, NM 87131, USA; remeny@salud.unm.edu; 3 Biomedical Libraries, Geisel School of Medicine at Dartmouth, Hanover, NH 03755, USA; pamela.bagley@dartmouth.edu (P.J.B.); heather.b.blunt@dartmouth.edu (H.B.B.); 4 New Hampshire Department of Environmental Services, Concord, NH 03302, USA; 5 Department of Obstetrics and Gynecology, Dartmouth Health, Lebanon, NH 03756, USA; 6 New London Hospital Association, New London, NH 03257, USA; 7 Department of Pediatrics, Geisel School of Medicine at Dartmouth, Dartmouth Cancer Center, Hanover, NH 03755, USA; 8 Department of Pediatric Hematology Oncology, Children’s Hospital Colorado, Aurora, CO 80045, USA; raymond.walston@childrenscolorado.org; 9 Department of Epidemiology, Geisel School of Medicine at Dartmouth, Dartmouth Cancer Center, Hanover, NH 03755, USA

**Keywords:** pediatric cancer, epidemiology, etiology

## Abstract

**Simple Summary:**

Much research has been conducted on the causes of childhood cancer, but many areas of uncertainty remain. We conducted a detailed search of recent studies investigating the causes of childhood cancer. Our findings, based on publications from 2014 to 2021, are summarized by topic in three separate papers: factors acting during childhood (Paper 1), factors relating to the parents and pregnancy (Paper 2), and factors relating to the environment (Paper 3). In this first paper, we summarize convincing evidence from studies that show increased childhood cancer risk associated with genetic cancer predisposition, birth defects, prior cancer, medical ionizing radiation, organ transplantation and exposure to cancer-causing viruses, and a reduced risk associated with some childhood vaccinations. Other factors discussed in this paper include the impact of the child’s diet, allergies, medications, and body mass index. This review of factors associated with childhood cancer may help evidence-based efforts to reduce cancer in our pediatric populations.

**Abstract:**

Purpose: To review the childhood risk factors for pediatric cancer (diagnosis before age 20). Methods: We conducted literature searches using Ovid Medline and Scopus to find primary research studies, review articles, and meta-analyses published from 2014 to 3 March 2021. Results: Strong evidence indicates that an array of genetic and epigenetic phenomena, structural birth defects, and chromosomal anomalies are associated with an increased risk of various childhood cancers. Increased risk is also associated with prior cancer, likely due to previous treatment agents and therapeutic ionizing radiation. Convincing evidence supports associations between several pediatric cancers and ionizing radiation, immunosuppression, and carcinogenic virus infection both in healthy children and in association with immune suppression following organ transplantation. Breastfeeding and a childhood diet rich in fruits and vegetables appears to reduce the risk of pediatric leukemia but the evidence is less strong. Childhood vaccination against carcinogenic viruses is associated with a lower risk of several cancers; there is less strong evidence that other childhood vaccinations more broadly may also lower risk. Ultraviolet (UV) radiation is associated with increased melanoma risk, although most melanomas following childhood UV exposure occur later, in adulthood. Evidence is weak or conflicting for the role of body mass index, other childhood infections, allergies, and certain treatments, including immunomodulator medications and human growth therapy.

## 1. Introduction

This manuscript is one of three reviews aimed at summarizing recent studies of risk factors for childhood cancers, generally defined as cancers diagnosed before the age of 20. In this installment, Part 1, we summarize evidence from studies investigating the role of child factors, including genetic predisposition, personal and behavioral characteristics, medical conditions, and medical/surgical treatments in relation to the risk of childhood cancer. Part 2 summarizes evidence from studies investigating parental exposures, and Part 3 summarizes the results of studies assessing environmental exposures. The topics covered by each paper in this series are shown in Table 1.

## 2. Materials and Methods

### 2.1. Search Strategy for Published Articles

Librarians (HBB, PJB) designed searches to find primary research studies and review articles. Searches were performed in Ovid Medline and Scopus from 2014 to 3 March 2021. No restrictions were applied. Search strategies were adjusted for the syntax of each database. Complete search strategies for each database are included in Supplementary Table S1.

To find primary studies, we searched for subject headings and text words representing four concepts: childhood cancer, cause, exposure, and measures of association. The childhood cancer concept included general terms for childhood cancer as well as specific cancer names listed by the International Classification of Childhood Cancer (3rd edition) [1]. Similarly, for the exposure concept, we used general words such as ‘exposure’ or ‘environmental’ as well as names of specific potential cancer-causing agents or classes of agents, e.g., diethylstilbestrol and flame retardants. For the cause concept, we included words such as ‘risk’, ‘etiology’, or ‘cluster’. To find studies that focused on an association between childhood cancer and exposure, we included words such as ‘association’, ‘rate ratios’, or ‘relative risk’.

A strategy was also designed to find review articles not captured by the primary study search. We searched for text words representing three concepts: childhood cancer, exposure, and cause. Review articles were identified from both searches if they were tagged by the database as a review or included ‘review’, ‘systematic’, ‘metaanalysis’, ‘meta-analysis’, or ‘scoping’ in the title.

Results from the research and review article searches were downloaded to separate EndNote libraries. After duplicates were removed, there were 3116 research articles and 746 review articles. Records were uploaded to Covidence for screening. Two independent researchers from the team screened every research article and removed those with irrelevant methods or results. In the case of a disagreement, an arbiter (JRR) made the final decision. Title and abstracts of the review articles were screened by one reviewer (HBB or PJB) to remove irrelevant results. After screening, 520 research articles and 462 review articles remained. The team defined a list of topics covered by these articles, and each paper was classified in terms of its relevance to one or more topics. Each major topic was assigned to a co-author for synthesis and summary of the papers that were relevant to it. The resulting manuscript was compiled, reviewed, and edited for content and continuity by a core team (AMR, RE, LT, JRR) before final review by the full team. A summary of these topics was created, describing the strength of the associations based on the reviewed literature (Table 2).

### 2.2. Descriptive Epidemiology

The age-standardized incidence of invasive cancers in children under twenty years of age in the United States has increased from 173 per million in 2003–2007 to 189 per million in 2013–2017, representing an increase of more than 9% over a decade [2]. Substantial geographic differences in incidence have been identified worldwide [3] and within the US, and there is a particularly high incidence in the northeastern United States [4,5]. Understanding the underlying causes of childhood cancers is necessary to explain these trends and identify preventive strategies, but the numbers of incident childhood cancers are relatively small, and etiologic research is difficult. Ecological studies of incidence and risk factors at the population level, including cancer cluster investigations, are limited by the lack of evidence of associations at the individual level. Meta-analyses can be useful to pool data from multiple smaller studies, provided that those studies are similar enough to merit data aggregation.

The incidence of childhood cancer varies substantially around the globe; worldwide in 2015, it was estimated that more than 360,000 cancers occurred in children aged under 15, many of them undiagnosed or undocumented [1,6]. The incidence of several cancer types varies by socioeconomic development; in more developed countries, there is a higher incidence of central nervous system tumors, neuroblastoma, leukemias, and germ cell tumors, whereas in less developed countries, the incidence of soft tissue sarcomas, lymphomas, and retinoblastoma is higher [6].

In the United States, approximately 15,000 children (age < 20) are diagnosed with cancer each year for an incidence of 173.7 cases per million children per year [5]. The incidence of first childhood cancers is 10% higher in boys than girls, and incidence peaks in children under 5 years old and 15–19-year-olds. The most common childhood cancers in the U.S. are leukemias (45.7 per million per year) > brain and central nervous system tumors (30.9) > lymphomas (26.2) > epithelial (17.8) > soft tissue (11.7) > germ cell/trophoblastic (11.0) > bone (8.6) > neuroblastoma (8.5) > renal (6.8) > retinoblastoma (3.2) > hepatic (2.3) [5]. The incidence of cancer subtypes can vary substantially by age. For example, in 0–4 and 5–9-year-olds, leukemias and central nervous system (CNS) cancers are most common, but leukemias are twice as common in 0–4-year-olds than in any other age group under 20. In 15–19-year-olds, lymphoma, germ cell tumors, and epithelial tumors (including melanoma) predominate [7]. Incidence also varies substantially by race/ethnicity (white > Hispanic > American Indian/Alaska Native > Asian/Pacific Islander > Black) and by region within the United States, with highest rates in the northeast and lowest in the south [4,5]. Higher rates have generally been associated with higher income or socioeconomic status (SES) [5,8,9]. SES is likely to be a surrogate for other environmental and/or parental occupational factors discussed below. The incidence of childhood cancer overall is increasing [10]. Although some argue that differences in cancer registration and case ascertainment account for some of the apparent increase [11], the different trends by subtype (such as decreasing skin cancer incidence, stable Hodgkin lymphoma incidence but increasing non-Hodgkin lymphoma incidence) seem to indicate real changes in incidence [12].

## 3. Genetic Predisposition

There have been tremendous advances in the understanding of genetics on childhood cancer over the past several decades; there is now strong evidence of the role of genetic predisposition in the etiology of some childhood cancer and emerging evidence of the role of epigenetics [13]. Unlike adult cancer, childhood cancer tends to have a lower burden of somatic mutations [14] and is heavily impacted by germline mutations in cancer predisposition genes [15,16,17]. Some of the most common syndromes include Li–Fraumeni Syndrome (LFS), Neurofibromatosis, Fanconi Anemia, DICER1 Syndrome, Multiple Endocrine Neoplasia, retinoblastoma predisposition, rhabdoid predisposition syndrome, APC polyposis, BRCA, and constitutional mismatch repair deficiency syndrome, among others [14,17,18]. The genes that are implicated in these various syndromes are generally classified as being related to DNA damage repair, cell cycle, and apoptosis regulation and are associated with a variety of cancer types. For example, Li–Fraumeni Syndrome is caused by the loss of function mutation of TP53, with over 250 mutations described. Patients with LFS are estimated to have a 50% chance of developing one malignant neoplasm by age 30 [19]. Soft tissue sarcoma and osteosarcoma are the most common neoplasms in children with germline TP53 mutation, but among children with low hypodiploid acute lymphoblastic leukemia (ALL), a particularly high risk phenotype, 91% had somatic TP53 mutation and nearly half of those patients had germline TP53 mutation [19,20]. Adrenocortical carcinoma (ACC) is a rare pediatric cancer, and up to 80% of children with sporadic ACC are found to have germline TP53 mutation [19]. Germline mutations in genes known to be involved with lymphocyte differentiation are associated with leukemiogenesis including PAX5, IZKF1, ETV6, RUNX1, and others [15,21].

Beyond known cancer predisposition genes, ongoing research explores the associations of common genetic variants and childhood cancer, but genome-wide association studies require large numbers of patients and candidate gene studies can be less definitive [16]. Despite this challenge, single nucleotide polymorphisms (SNPs) of interest have been identified for multiple pediatric concerns including brain tumors, ALL, osteosarcoma, and neuroblastoma [21,22,23,24,25,26]. More research is needed to determine whether SNPs interact with environmental exposures to influence childhood cancer risk [25]. Epigenetics, including DNA methylation, histone modification, and chromatin remodeling, also plays an important role in childhood cancer. Beckwith–Wiedemann Syndrome (BWS), an overgrowth syndrome, arises from a variety of epigenetic imprinting methylation defects at 11p15 and is associated with an increased risk for several childhood tumors, particularly Wilms tumor and hepatoblastoma. The risk of tumor development may be as high as 20–30% for patients with ICR1 gain of methylation [27,28,29]. Epigenetic changes also seem to be the drivers for multiple aggressive pediatric malignancies including alterations of the SWI/SNF chromatin remodeling complex in rhabdoid tumors and histone mutation in diffuse intrinsic pontine glioma [14,30,31]. How epigenetic mutations arise remains unclear, and it is postulated that environmental exposures may have an impact on DNA methylation [27,32].

## 4. Birth Defects

There is strong evidence of associations between birth defects and an increased risk of childhood cancer. A systematic review in 2017 demonstrated a 1.8–3.05-fold increased risk of childhood cancer associated with any or major birth defects based on analyzed cohort studies [33], and a multistate, registry linkage study of more than 10 million children reported that the risk of cancer before age 18 was 2.5 times as high in children with non-chromosomal birth defects and 11.6 times as high in children with chromosomal abnormalities, when compared with children without such abnormalities [34]. Further, the more birth defects that are present, the greater the risk of childhood cancer [34]. The most well established is the association of Down Syndrome Trisomy 21 with leukemia. Down syndrome increases the risk of ALL 20-fold and the risk of acute megakaryocytic leukemia (AML) 500-fold [21,33]. Other rarer chromosomal disorders also predispose to childhood cancer (e.g., Klinefelter syndrome and malignant germ cell tumors) [18]. In terms of other specific structural birth defects, anomalies of the nervous system are associated with an increased risk for brain tumors [33,35]. Other suggested associations have been noted between a variety of birth defects and neuroblastoma, as well as between genitourinary abnormalities and hepatoblastoma [16,33,34].

## 5. Prior Cancer and Cancer Treatments

There is strong evidence that individuals of any age who are treated for cancer are more likely to experience additional cancers [36]. Treatment for childhood cancer is a recognized risk for the development of subsequent primary malignant neoplasm (SMN), with a 4- to 6-fold increased risk of malignancy over 20–40 years of follow-up across several cohort studies [37,38]. This may result from exposure to ionizing radiation through radiotherapy, medical imaging such as CT scans, and through chemotherapy include alkylating agents, topoisomerase inhibitors, and anthracycline [36]. There has been a decrease in the proportions of patients developing an SMN within the subsequent 15 years from 2.1% for diagnoses in the 1970s to 1.3% for diagnoses in the 1990s (*p* < 0.001), which is thought to be due to the considerable attention paid to reducing exposure where possible to radiation therapy and specific chemotherapeutic agents [38,39].

## 6. Medical Ionizing Radiation

A strong link between ionizing radiation and childhood cancer has been recognized for more than 75 years. The International Agency for Research on Cancer (IARC) classifies ionizing radiation as a group 1 carcinogen, ‘carcinogenic to humans’ [40]. In 1972, the National Academy of Sciences (NAS) was asked to assess national radiation protection guidelines because of the recognition of the increased cancer risk following atomic bomb radiation exposures, the growth of nuclear power, and the development of cancers after irradiation for a variety of conditions in childhood including thymic enlargement [41,42]. In the assessment published by the NAS in 1972, the average dose of background radiation in the United States was estimated to be 100 mrem (1 mSv) per year [42], a figure that by 2006 had increased to 6.2 mSv [43]. Non-iatrogenic sources of radiation will be discussed in Part 3.

Recent research has focused on the importance of iatrogenic exposure to ionizing radiation through medical imaging. In excess of 91 million computerized tomography (CT) scans are performed in the United States annually—22 per 100 inhabitants [44]. A dose-dependent association has been reported between radiation from CT scans in childhood and the risks of leukemia and of brain cancer [45]. A meta-analysis identified no significant association between childhood cancer risk and pre- or postnatal X-rays or prenatal CT scans, but CT scans in childhood were associated with an excess risk of leukemia (pooled excess relative risk ERR_pooled_ 26.9%; 95% CI: 2.7–57.1) and brain tumors (ERR_pooled_ 9.1%; 95% CI: 5.2–13.1) [46]. An Australian retrospective cohort study of 10.9 million children followed for a mean of 9.5 years showed an excess cancer risk of 24% among those who had a CT scan before age 20, with the greatest risk when CT scans were performed before age 5 (IRR 1.35; 1.25–1.45) [47]. It has been estimated that CT scans of the head conducted in 1000 children through age 5 would cause one excess cancer during a typical lifespan [48]. A similar risk was reported by Miglioretti who estimated that 4870 cancers throughout the lifespan may result from the 4 million CT scans conducted during childhood in the United States [49].

It is unclear whether regional variation in CT scan use may account for part of the geographic variation in US childhood cancer incidence. In multivariable analyses, Lodwick found that CT scan use appears to be the highest in children with the highest median household income, in those with private insurance, significantly higher in boys than girls, and the lowest in the Northeast [50] where pediatric cancer incidence is highest [4,5,51,52]. Additional studies have shown the more frequent use of CT scans among white and privately insured children [53,54,55,56,57], and parental anxiety has been cited as a possible reason for some of the excess CT use in white children [57]. Children treated for trauma (2010–2013) in Arkansas received twice as much radiation when treated at non-pediatric hospitals [58], and such a difference might pose a risk in rural areas with poorer access to specialist pediatric hospitals.

In the United States, thyroid cancer incidence in children aged <20 increased by more than 10% per year from 2006 to 2012 but has since shown signs of slowing [12]. Stefan et al. reviewed data on increases seen after the detonation of nuclear bombs in Japan and in the Marshall islands and after the nuclear power plant accident in Chernobyl in 1986. Their review argues that, whereas much of the increase in thyroid cancer being seen in adults may be due to the increased use of medical imaging leading to incidental findings, the increases seen elsewhere in children are real and are not attributable to overdiagnosis [59].

## 7. Ultraviolet (UV) Light

Sunburn and exposure to sunlight during childhood are risk factors for melanoma and other skin cancers that are diagnosed throughout life [60]. Exposure to environmental UV radiation may be due to sunlight, sunlamps, and tanning beds. The spectrum of UV light to which humans are exposed includes UVA (typically non-ionizing) and UVB (typically ionizing). UVB has sufficient energy to damage cells and DNA and may account for most skin cancers, but UVA may also contribute to cancer risk.

Exposure to ultraviolet radiation through sun exposure and tanning beds is a well-known risk factor for adult skin cancers and melanomas; efforts to reduce the risk of these malignancies have included public health education campaigns regarding sun exposure and the use of tanning beds and the associated cancer risk [61]. Several European countries and most US states have restricted or banned the use of tanning beds by minors [62]. Melanoma incidence in children increased for nearly three decades prior to 2004, but recent rates have declined by an average of 3–4% per year [12]. Older ecologic studies offered evidence of an association between pediatric melanoma and ambient UV radiation [60], and a large SEER-based study found an association between ambient sun exposure using an EPA irradiance measure and melanoma in children and young adults (age < 25) [63]. Although these decreases may reflect the influence of public health messaging regarding sun exposure, most earlier case–control studies of pediatric melanoma have found associations with moles and constitutional characteristics rather than sun exposure [60]. However, in a 2019 case–control study, Wojcik reported that the risk of melanoma among 15–19-year-olds was significantly increased among children born in areas with ambient daily UV above the lowest quartile, after adjustment for other factors including birth weight [64].

Recent initiatives have attempted to reduce the use of indoor tanning by minors and to provide education on the importance of sun protection in childhood [61]. The incidence of melanoma in children increased from 1975 to 2004, but the subsequent decrease of 3.4% annually [12] is an encouraging trend.

## 8. Organ Transplantation

There is strong evidence that immunosuppression following organ transplant is associated with an increased risk of cancer. Cancer incidence in solid organ tissue recipients is 19-fold higher than in the general population [65], and as many as one-third of pediatric solid organ transplant recipients will develop cancer within 30 years [66]. The increased risk after transplant is secondary to immunosuppression and oncogenic infections which can be directly transmitted via solid organ donors [66,67]. As long-term survival has improved for pediatric recipients of solid organ transplantation, the risk of post-transplant malignancies has also increased. Solid organ transplant recipients are at a higher risk for several cancers including post-transplant lymphoproliferative disorders (PTLD), skin cancers, angiosarcomas, lymphomas, and myelomas [65,66]. The highest risk is seen with intestinal transplant, which is thought to be due to the gastrointestinal-associated lymphatic tissue carriage of viral infections such as oncogenic Epstein–Barr virus [65] (See Section 12).

## 9. Medications in Childhood

### 9.1. Human Growth Hormone

Studies of growth hormone (GH) can be divided into those of children without a history of cancer and those involving pediatric cancer patients whose treatment left them with growth hormone deficiency. The Pediatric Endocrine Society Drug and Therapeutics Committee concluded that in children without known risk factors for malignancy, GH therapy could safely be given without increasing the risk of cancer [68]. However, the use of GH in cancer survivors may increase the risk of second cancers; it can be used in those children provided the risks are understood and appropriate monitoring is conducted [69]. In a study of meningioma after growth hormone treatment, the standardized incidence ratio (SIR) associated with prior CNS tumor and GH treatment was 5.3 (95% CI 3.7–7.7) indicating a more than 5-fold increase in risk, whereas the SIR after non-cancer GH treatment was not significantly elevated, suggesting that the main increase in risk arises from the initial treatment for cancer rather than the GH itself [70].

### 9.2. Immunomodulatory Medications

The FDA has issued several cancer warnings and a cancer surveillance requirement for patients aged <30 treated with tumor necrosis factor (TNF) inhibitors (TNF-I), although the initial FDA report was criticized for methodological reasons [71,72,73,74,75]. Juvenile Idiopathic Arthritis (JIA) and inflammatory bowel disease (IBD) are the most common indication for the use of TNF-I in children; it is uncertain whether these conditions carry an intrinsically higher risk of cancer, or whether the risk is elevated by immunomodulatory medications such as methotrexate (MTX) and TNF-I used to treat the condition. Dulai reviewed 65 studies of TNF-I in IBD and found no significant association (SIR 3.5; 0.35–19.6) based on only two cases of lymphoma [76]. Simard used the Swedish registry to compare cancer incidence in a large cohort of children with JIA and controls with attention deficit hyperactivity disorder; no significantly increased cancer risk was seen with JIA from 1969 to 1986, but from 1987 to 2007, the RR was 2.3 (95% CI 1.2–4.4) perhaps because of methotrexate use in the later period [77]. The consensus seems to be that JIA is associated with an increased risk of childhood cancer but that there is no convincing strong role of TNF-I [78]. Mannion’s assessment of the literature indicated a 2–4-fold increased risk of cancer in children with JIA. Although there was no evidence to confirm a lack of association between MTX or TNF-I and cancer risk in JIA, the studies were small or lacked follow-up [73]. There is evidence from population-based studies that IBD treated with thiopurines such as azathioprine for at least a year is associated with a 6-fold increased risk of lymphoma [79]. In particular, a very aggressive but rare hepatosplenic T-cell lymphoma was noted in IBD patients, mostly in males; Kotlyar reported that 36 of 200 cases of this type of lymphoma reported in the literature since 1996 had occurred in children with IBD [80].

## 10. Diet

There is evidence that children’s diets could be associated with childhood cancer risk. We consider two aspects of diet: breastfeeding and childhood diet.

### 10.1. Breast Feeding

Breastfeeding has been found to be protective against many chronic illnesses, including obesity and diabetes, and to have significant benefits for infant immunity, reducing mortality from common infections including respiratory infections [81]. Recently, there has been significant interest in evaluating the impact of breastfeeding on the risk of childhood cancer. A meta-analysis of 17 case–control studies showed breastfeeding for 6 months or longer was associated with a 20% lower risk for childhood leukemia (17 studies, OR 0.80; 95% CI, 0.72–0.90) compared to no breastfeeding or a shorter period of breastfeeding [82]. The authors also assessed 14 case–control studies that compared ever versus never breastfeeding, finding that some breastfeeding was associated with a 9% lower risk for childhood leukemia (14 studies, OR 0.91; 95% CI, 0.80–1.04). In a more recent meta-analysis, the most protective association for leukemia was seen for a breast feeding duration of 9.6 months (33 studies, OR, 0.66; 95% CI 0.62–0.70), and for neuroblastoma, ever breastfeeding was also protective (4 studies, OR 0.61; 95% CI 0.44–0.83), but no associations were seen with other cancer types [83]. A case–control study found a markedly reduced risk of rhabdomyosarcoma in children who were breastfed for at least 12 months (OR: 0.36; 0.18, 0.70) [84]. The impact on other cancer subtypes is less clear [13].

### 10.2. Childhood Diet

Several studies indicate that diet during childhood is an important factor in childhood cancer risk. An increased risk of leukemia and lymphoma has been linked to the consumption of processed foods such as candy or cured meats three or more times per week (OR 3.03; 1.37–6.71) [85]. Children up to age 2, whose parent reported that the child ate no vegetables, also had increased risk for ALL and AML (OR 2.40; 1.07–5.40) [85]. A meta-analysis showed that leukemia risk was inversely associated with diets high in fruit and vegetables and higher for diets high in processed meat products; however, neither of these results were statistically significant [86]. The association between maternal diet before and during pregnancy and childhood cancer will be discussed in Paper 2. Specht et al. examined the impact of the legislation in Denmark to ban industrial trans-fatty acids from foodstuffs and found no clear impact on the incidence of leukemia or lymphoma, although they could not exclude such an association [87].

## 11. Body Mass Index

Because there is an epidemic of childhood overweight and obesity, it is critical to understand its impact on cancer both in childhood and adulthood [88]. While there are suggestions that obesity in childhood may impact childhood cancer prognosis [89], there is little research on any etiologic connection for the risk of cancer.

## 12. Infections

### 12.1. Carcinogenic Viruses

The IARC has classified several common infectious agents as carcinogenic, including human papillomavirus (HPV), hepatitis B and C viruses, human immunodeficiency virus (HIV), human herpes virus 8 (HHV8), and Epstein–Barr virus (HHV4) [40]. The first virus found to be associated with cancer was Epstein–Barr virus (EBV), which causes infectious mononucleosis (“mono”), and Burkitt’s lymphoma, which is an aggressive lymphoma that is common in malaria-endemic areas where it comprises more than half of childhood cancers. It is thought that co-infection with both malaria and EBV causes the conditions needed for carcinogenesis, because EBV alone does not seem to be sufficient. It may be that Plasmodium falciparum infection affects the B-cells that are latently infected by EBV such that EBV is reactivated; an alternative theory supposes that T-cell function is impaired by *P. falciparum* infection, rendering the body vulnerable to oncogenic effects of EBV (reviewed by Moorman [90]). In areas of endemic Burkitt’s lymphoma, seasonal and geographic variation are seen that relate to malaria prevalence, consistent with this two-infection etiology, and Burkitt’s lymphoma is also seen in the immune suppression of untreated HIV infection in the United States, again indicating the importance of immunity in the prevention of cancer (reviewed by Jayajanani [91]).

In the United States, 30% of patients with Hodgkin’s lymphomas test positive for EBV, and studies have shown an association with prior infectious mononucleosis (reviewed by Hjalgrim and Jarrett [92]). In the pre-diagnostic serum of adults who went on to develop EBV-positive and EBV-negative Hodgkin’s lymphoma, differences have been observed in the immune response to EBV infection [93]. Lymphoma in the setting of immune deficiency in HIV and transplantation also tends to be EBV-positive [92]. It has been estimated that EBV accounts for more than a quarter million new cancers annually across all ages worldwide [94].

Among the other examples of carcinogenic viruses, HHV8 causes Kaposi’s sarcoma (KS) most often in states of immune deficiency, including in untreated HIV infection. In the United States, there are 100–200 cases of perinatal childhood HIV diagnosed annually; this number is relatively low because the prevention of perinatal transmission by antiretroviral treatment is very effective [95]. In the setting of poor immunity, the risk of other types of cancer increases, including non-Hodgkin’s lymphoma (see review by Singh [96]).

### 12.2. Exposure to Common Infections in Childhood

Infections may also be important in childhood cancer by different mechanisms. It has been suggested that exposure to common infections in early childhood may be important for the developing immune system and for susceptibility to cancer through a robust immune response. Greaves proposed that later infection may actually trigger the development of leukemia [97], and this fits with his two-step model of leukemiogenesis, in which a prenatal event is followed by a second trigger in a susceptible child—in this case, an infection [98]. Studies examining the role of infection seek to understand the role of the impact of the immunological response to infection both in prenatal and postnatal development as well as those with immunological dysregulation in childhood and the influence on pediatric cancer risk. A nationwide study in Taiwan found that infection with enterovirus more than halved the risk of childhood ALL and AML [99]. Rudant showed a lower risk of ALL in children who attended daycare in the first year of life (OR 0.77; 95% 0.71–0.84), with a trend for the lowest risk with the earliest attendance [98]. Rudant also reported that among children who were not breastfed, repeated early common infections were associated with lower Hodgkin lymphoma risk (OR 0.3; 0.2–0.7) [100]. Lupo found a reduced risk of rhabdomyosarcoma in children who attended daycare before entering kindergarten [101]. Evidence for infectious etiology in brain cancer comes from studies of birth order and number of siblings, day care attendance, and cancer clusters/population mixing. It has been suggested that infections in early childhood lower the risk, and those in later childhood increase the risk or actually trigger cancer development; future studies may need to stratify by age at cancer onset [102]. These concepts could potentially tie together evidence about the protective effect of vaccinations and of early childhood infections and population mixing as a source of infections in both early and late childhood. For example, a leukemia cluster in a new Scottish town led to the hypothesis that “population mixing”, which brought people together in the new community, led to an increase in common childhood infections [103].

## 13. Vaccinations

Several papers have indicated that childhood vaccination may be protective against some types of childhood cancer. A study in Taiwan also showed that hepatitis B vaccination is associated with reduced risk of hepatocellular carcinoma (HCC) in children and young adults (OR 0.18; 0.10–0.32) for all age ranges and stratified by gender [104]. The prevention of chronic infection, and hence cancer, is a specific goal of vaccination against this carcinogenic virus. However, there is evidence of a more general benefit of vaccination even when the target is not a carcinogenic virus. A 2020 meta-analysis showed a 38% lower risk of childhood cancer in children who received three vaccinations of any type (OR 0.62; 0.46–0.85), significant protective associations for leukemia with bacillus Calmette–Guérin vaccination (BCG vaccine to prevent tuberculosis), and Hemophilus influenzae type b vaccination in relation to leukemia [105]. In a case–control study, incomplete immunization was associated with a 5-fold higher risk of rhabdomyosarcoma (OR 5.30; CI 95% 2.47–11.33), and significant trends were seen with incomplete and no diphtheria, tetanus, and pertussis vaccination (*p* = 0.022) and with incomplete or no oral polio vaccination (*p* = 0.026) [106].

## 14. Allergies

The role that allergy plays in the pathophysiology of the emergence of cancer in children is unclear. A case–control study found a reduced risk of rhabdomyosarcoma in children with allergies (OR 0.60; 95% CI 0.41–0.87) [101]. A recent case–control study and meta-analysis of 12 studies examined the association between allergies and ALL with mixed results; overall, there was not convincing evidence of an association, and an apparent protective association with hay fever might have been explained by the misclassification of mild upper respiratory tract infections [107]. For brain cancers, some studies have suggested a protective effect of allergies but others do not (reviewed by Johnson [102]).

## 15. Limitations

This review provides summary evidence from papers published within a short timeframe (2014 to early 2021) and therefore excludes older and more recent evidence. Further limitations in this field generally include the many methodological issues that hinder research in pediatric cancer, including biases in the measurement of exposures, recall of exposures, selection of participants included in studies, selection of controls where applicable, and small sample sizes causing type II error.

## 16. Conclusions

We found strong evidence of associations between pediatric cancer and various genetic and epigenetic phenomena, structural birth defects and chromosomal anomalies, previous cancer (likely due to previous treatment agents and therapeutic ionizing radiation), ionizing radiation, immunosuppression, carcinogenic virus infection, and organ transplantation. Childhood diets rich in fruits and vegetables appear to reduce the risk of pediatric leukemia, and maternal diet during pregnancy showed a similar protective association. Childhood vaccination is associated with lower risk of several cancers. Ultraviolet (UV) radiation is associated with an increased childhood melanoma risk, although most melanomas following childhood UV exposure occur later, in adulthood. The evidence is weak or conflicting for associations between pediatric cancer and body mass index, childhood infections, allergies, immunomodulator medications, and human growth therapy.

## Figures and Tables

**Table 1:** Content of the three parts of this review.

Paper 1 Child Factors	Paper 2 Parental and Pregnancy Factors	Paper 3 Environmental Factors
Genetic predisposition	Alcohol	Outdoor pollution
Birth defects	Smoking	Indoor pollution
Prior cancer and associated treatments	Diet and vitamins	Occupational exposures:
Medical ionizing radiation	Caffeine	- Benzene
Ultraviolet (UV) light	Maternal age	- Diesel
Organ transplantation	Maternal diabetes	- Agricultural animals
Medications in childhood	Maternal obesity	- Agricultural pesticides
Diet and breastfeeding	Birth and obstetric history	- Other
Body mass index	Birth weight	Ionizing radiation:
Infections	Gestational age	- Nuclear power plants
Vaccinations	Multiple gestation	- Radon
Allergies	Birth order	Non-ionizing radiation
	Cesarean section and instrumental delivery	
	Assistive reproductive technologies	
	Medications during pregnancy	
	Medical ionizing radiation	

**Table 2:** Summary of the strength of evidence associating child characteristics and exposures with childhood cancer.

Exposure	Notes
Strong evidence of association with childhood cancer	
Genetics	Germline cancer predisposition genes are strongly associated with an increased risk of multiple childhood cancers. There is also increasing research into the impact of more common genetic variants and epigenetics.
Birth defects	Major birth defects and some chromosomal syndromes are strongly associated with an increased risk of multiple childhood cancers.
Prior cancer and cancer treatments	Childhood cancer treatment is associated with a significant risk of developing a subsequent primary cancer.
Medical ionizing radiation	There is a strong link between CT scans in childhood and an increased risk of childhood cancer, particularly leukemia and brain cancer.
Ultraviolet light	Exposure to UV light in childhood is associated with a significant risk of melanoma in later life. Public health interventions to reduce indoor tanning by minors is associated with decreased cancer incidence.
Organ transplantation	Immunosuppression after solid organ transplant is associated with a significantly increased risk for several childhood cancers.
Vaccinations	Vaccination against certain carcinogenic viruses is strongly associated with a decreased childhood cancer risk. There is more limited evidence that childhood vaccinations (against non-carcinogenic viruses) more broadly may decrease cancer risk.
Infections	Certain carcinogenic viruses (e.g., Epstein–Barr virus) are strongly associated with childhood cancer. There is limited evidence that exposure to common childhood infections may decrease childhood cancer risk.
Diet and breastfeeding	Breastfeeding is associated with a lower risk of leukemia, and possibly rhabdomyosarcoma, but evidence is lacking for other cancer types. There was some evidence for decreased leukemia risk associated with improved diet quality and a higher cancer risk associated with lower diet quality.
Mixed evidence of association with childhood cancer	
Allergies	Rhabdomyosarcoma is less common in children with allergies, but the evidence on associations between allergies and other childhood cancer risk is mixed.
Weak or no evidence of association with childhood cancer	
Medications in childhood	Human growth hormone was not associated with a significantly increased risk of a first childhood cancer, although the risk may be different when used in childhood cancer survivors. Studies evaluating immunomodulatory agents and childhood cancer risk have been methodologically challenged and while it seems underlying inflammatory conditions may be associated with an increased risk for childhood cancer, the role of medications is not clearly established.
Body mass index	There was too little research to draw conclusions on the impact of childhood obesity on childhood cancer risk.

## Supplementary Materials

The following supporting information can be downloaded at: https://www.mdpi.com/article/10.3390/cancers16071297/s1, Table S1: Search strategies.
