# Correction: Ricci et al. Causes of Childhood Cancer: A Review of the Recent Literature: Part I—Childhood Factors. *Cancers* 2024, *16*, 1297

**DOI:** 10.3390/cancers18081231

**Published:** 2026-04-14

**Authors:** Angela M. Ricci, Rebecca T. Emeny, Pamela J. Bagley, Heather B. Blunt, Mary E. Butow, Alexandra Morgan, Jennifer A. Alford-Teaster, Linda Titus, Raymond R. Walston, Judy R. Rees

**Affiliations:** 1 Department of Pediatrics, Division of Pediatric Hematology-Oncology, Dartmouth Health Children’s, Lebanon, NH 03756, USA; 2 Department of Internal Medicine, Division of Molecular Medicine, UNM Comprehensive Cancer Center, Cancer Control & Population Sciences Research Program, University of New Mexico Health Sciences, Albuquerque, NM 87131, USA; 3 Biomedical Libraries, Geisel School of Medicine at Dartmouth, Hanover, NH 03755, USA; pamela.bagley@dartmouth.edu (P.J.B.); heather.b.blunt@dartmouth.edu (H.B.B.); 4 New Hampshire Department of Environmental Services, Concord, NH 03302, USA; 5 Department of Obstetrics and Gynecology, Dartmouth Health, Lebanon, NH 03756, USA; 6 New London Hospital Association, New London, NH 03257, USA; 7 Department of Pediatrics, Geisel School of Medicine at Dartmouth, Dartmouth Cancer Center, Hanover, NH 03755, USA; 8 Department of Pediatric Hematology Oncology, Children’s Hospital Colorado, Aurora, CO 80045, USA; raymond.walston@childrenscolorado.org; 9 Department of Epidemiology, Geisel School of Medicine at Dartmouth, Dartmouth Cancer Center, Hanover, NH 03755, USA

## Error in Table

In the original publication [1], there was a mistake in Table 2 as published. Table 2 incorrectly states that there is mixed evidence of an association between childhood cancer and diet and breastfeeding, and strong evidence of an association between childhood cancer and allergies. It should have stated that there was strong evidence for diet and breastfeeding and mixed evidence for allergies. The corrected Table 2 appears below. The authors state that the scientific conclusions are unaffected. This correction was approved by the Academic Editor. The original publication has also been updated.

## Figures and Tables

**Table 2:** Summary of the strength of evidence associating child characteristics and exposures with childhood cancer.

Exposure	Notes
Strong evidence of association with childhood cancer	
Genetics	Germline cancer predisposition genes are strongly associated with an increased risk of multiple childhood cancers. There is also increasing research into the impact of more common genetic variants and epigenetics.
Birth defects	Major birth defects and some chromosomal syndromes are strongly associated with an increased risk of multiple childhood cancers.
Prior cancer and cancer treatments	Childhood cancer treatment is associated with a significant risk of developing a subsequent primary cancer.
Medical ionizing radiation	There is a strong link between CT scans in childhood and an increased risk of childhood cancer, particularly leukemia and brain cancer.
Ultraviolet light	Exposure to UV light in childhood is associated with a significant risk of melanoma in later life. Public health interventions to reduce indoor tanning by minors is associated with decreased cancer incidence.
Organ transplantation	Immunosuppression after solid organ transplant is associated with a significantly increased risk for several childhood cancers.
Vaccinations	Vaccination against certain carcinogenic viruses is strongly associated with a decreased childhood cancer risk. There is more limited evidence that childhood vaccinations (against non-carcinogenic viruses) more broadly may decrease cancer risk.
Infections	Certain carcinogenic viruses (e.g., Epstein–Barr virus) are strongly associated with childhood cancer. There is limited evidence that exposure to common childhood infections may decrease childhood cancer risk.
Diet and breastfeeding	Breastfeeding is associated with a lower risk of leukemia, and possibly rhabdomyosarcoma, but evidence is lacking for other cancer types. There was some evidence for decreased leukemia risk associated with improved diet quality and a higher cancer risk associated with lower diet quality.
Mixed evidence of association with childhood cancer	
Allergies	Rhabdomyosarcoma is less common in children with allergies, but the evidence on associations between allergies and other childhood cancer risk is mixed.
Weak or no evidence of association with childhood cancer	
Medications in childhood	Human growth hormone was not associated with a significantly increased risk of a first childhood cancer, although the risk may be different when used in childhood cancer survivors. Studies evaluating immunomodulatory agents and childhood cancer risk have been methodologically challenged and while it seems underlying inflammatory conditions may be associated with an increased risk for childhood cancer, the role of medications is not clearly established.
Body mass index	There was too little research to draw conclusions on the impact of childhood obesity on childhood cancer risk.

## References

[1] Ricci A.M., Emeny R.T., Bagley P.J., Blunt H.B., Butow M.E., Morgan A., Alford-Teaster J.A., Titus L., Walston R.R., Rees J.R. (2024). Causes of Childhood Cancer: A Review of the Recent Literature: Part I—Childhood Factors. Cancers.

