# Supplementary material for: Causes of Childhood Cancer: A Review of the Recent Literature: Part I—Childhood Factors
Source: Cancers (Basel). 2024 Mar 27;16(7):1297. doi: 10.3390/cancers16071297 (PMC11011156; doi:10.3390/cancers16071297)
Supplement: Supplementary file 1 [file cancers-16-01297-s001.zip › cancers-2911154-supplementary.pdf]

Supplementary Table S1: Search Strategies

Database	Platform	Years covered	Date conducted	# results
Medline	Ovid	2014-current	20210317	Research 1668<br>Review 400
Scopus	Elsevier	2014-current	20210317	Research 2917<br>Review 667
Total				5652
With duplicates removed				Research 3116<br>Review 746

Medline (OVID)

Database(s): **Ovid MEDLINE(R) and Epub Ahead of Print, In-Process, In-Data-Review & Other Non-Indexed Citations and Daily** 1946 to March 17, 2021

#	Searches	Results
1	(exp *Neoplasms/ci, ep, et and (adolescen* or child* or infan* or neonat* or p?ediatric* or prenatal).ti,kf.) or ((adenoma* or astrocytoma* or Blastoma* or Cancer* or Carcinoma* or Chondrosarcoma* or chordoma* or craniopharyngioma* or Ependymoma* or Fibrosarcoma* or gangliocytoma* or ganglioglioma* or ganglioma* or Ganglioneuroblastoma* or germinoma* or Glioblastoma* or Glioma* or hemangioblastoma* or Hepatoblastoma* or ILCRs or Leuk?emia* or Lymphoma* or Malignan* or Medulloblastoma* or Melanoma* or meningioma* or Myelodysplastic syndrome* or Myeloproliferative disease* or Neoplasm* or nephroblastoma* or Neuroblastoma or neuroma* or oligodendroglioma* or Oncology or Osteosarcoma* or pineoblastoma* or pineocytoma* or Retinoblastoma* or Rhabdomyosarcoma* or RMS or Sarcoma* or schwannoma* or Tumor* or Tumour*) adj4 (adolescen* or child* or infan* or neonat* or p?ediatric* or prenatal)).ti,ab,kf.	105795
2	exp *Risk/ or *Space-Time Clustering/ or exp *Neoplasms/et or (Risk* or Etiolog* or Aetiolog* or Caus* or Cluster* or epidemiol* or Protect*).ti,kf. or (risk* adj8 (adenoma* or astrocytoma* or Blastoma* or Cancer* or Carcinoma* or Childhood all or Chondrosarcoma* or chordoma* or craniopharyngioma* or Ependymoma* or Fibrosarcoma* or gangliocytoma* or ganglioglioma* or ganglioma* or Ganglioneuroblastoma* or germinoma* or Glioblastoma* or Glioma* or hemangioblastoma* or Hepatoblastoma* or ILCRs or Leuk?emia* or Lymphoma* or Malignan* or Medulloblastoma* or Melanoma* or meningioma* or Myelodysplastic syndrome* or Myeloproliferative disease* or Neoplasm* or nephroblastoma* or Neuroblastoma or neuroma* or oligodendroglioma* or Oncology or Osteosarcoma* or pineoblastoma* or pineocytoma* or Retinoblastoma* or Rhabdomyosarcoma* or RMS or Sarcoma* or schwannoma* or Tumor* or Tumour*)).ab.	1625521

3	exp Agrochemicals/ or exp Carcinogens/ or exp Environmental Pollutants/ or exp Environmental Pollution/ or exp Radiologic Health/ or exp Radiation/ae or (Agrochemical* or Arsenic or Assisted reproductive technolog* or Birth defect* or Birth order* or Birthweight or Birth weight or Bisphenol A or BPA or Breastfeed* or Breast-feed* or Breast milk or Carcinogen* or Chemical* or Circadian or Cluster* or Contamin* or Day care or Daycare or DES or Diethylstilbestrol or Diethylstilboestrol or Diesel or Diagnostic x-ray or Dioxin or Dye* or ecigarette* or e-cigarette* or Electric transmission or Electromagnetic fields or Endocrine disruptor* or Environment* or Epigenetic* or Exposure* or Fertility treatment* or Flame retardant* or Fluorocarbons or Food* or Gasoline or Gene-environment* or Geographic* or Hazardous waste or Heavier at birth or Human milk or Hydrocarbon* or In vitro fertilization or Infection* or Insecticide* or Irradiation or IVF or Maternal or Medical imaging or Nuclear plant* or Nuclear reactor* or Obesity or Parental or Paternal or Perfluorooctanoic acid or PFOA or Pesticide* or Petrochemical or Petroleum or Perfluorooctanesulfonic acid or PFAS or PFOS or Phenol* or Phototherapy or Plastic* or Pollut* or Polycyclic Aromatic Hydrocarbons or Preeclampsia or Pre-eclampsia or Preterm or Radiation or Radon or Season of birth or Smok* or Soil or Solvent* or Sunscreen or Traffic or Vehicle emissions or Vaping or Water).ti,ab,kf.	7542432
4	Case-Control Studies/ or odds ratio/ or (association* or associated or attributable risk* or case control or odds ratio* or rate ratio* or regression or Relative risk or risk ratio*).ti,ab,kf.	5261769
5	1 and 2 and 3 and 4	4865
6	limit 5 to yr="2014 -Current"	1973
7	limit 6 to "review articles"	237
8	(systematic or meta analysis or metaanalysis or scoping or review).ti.	610508
9	6 and 8	138
10	((adenoma* or astrocytoma* or Blastoma* or Cancer* or Carcinoma* or Chondrosarcoma* or chordoma* or craniopharyngioma* or Ependymoma* or Fibrosarcoma* or gangliocytoma* or ganglioglioma* or ganglioma* or Ganglioneuroblastoma* or germinoma* or Glioblastoma* or Glioma* or hemangioblastoma* or Hepatoblastoma* or ILCRs or Leuk?emia* or Lymphoma* or Malignan* or Medulloblastoma* or Melanoma* or meningioma* or Myelodysplastic syndrome* or Myeloproliferative disease* or Neoplasm* or nephroblastoma* or Neuroblastoma or neuroma* or oligodendroglioma* or Oncology or Osteosarcoma* or pineoblastoma* or pineocytoma* or Retinoblastoma* or Rhabdomyosarcoma* or RMS or Sarcoma* or schwannoma* or Tumor* or Tumour*) adj4 (adolescen* or child* or infan* or neonat* or p?ediatric* or prenatal) adj10 (Risk* or Etiolog* or Aetiolog* or Caus* or Circadian or Cluster* or epidemiol* or Protect*) **adj10** (Agrochemical* or Arsenic or Assisted reproductive technolog* or Birth defect* or Birth order* or Birthweight or Birth weight or Bisphenol A or BPA or Breastfeed* or Breast-feed* or Breast milk or Carcinogen* or Chemical* or Cluster* or Contamin* or Day care or Daycare or DES or Diethylstilbestrol or Diethylstilboestrol or Diesel or Diagnostic x-ray or Dioxin or Dye* or ecigarette* or e-cigarette* or Electric transmission or Electromagnetic fields or Endocrine disruptor* or Environment* or Epigenetic* or Exposure* or Fertility treatment* or Flame retardant* or Fluorocarbons or Food* or Gasoline or Gene-environment* or Geographic* or Hazardous waste or Heavier at birth or Human milk or Hydrocarbon* or In vitro fertilization or Infection* or Insecticide* or Irradiation or IVF or Maternal or Medical imaging or Nuclear plant* or Nuclear reactor* or Obesity or Parental or Paternal or Perfluorooctanoic acid or PFOA or Pesticide* or Petrochemical or Petroleum or Perfluorooctanesulfonic acid or PFAS or PFOS or Phenol* or Phototherapy or Plastic* or Pollut* or Polycyclic Aromatic Hydrocarbons or Preeclampsia or Pre-eclampsia or Preterm or Radiation or Radon or Season of birth or Smok* or Soil or Solvent* or Sunscreen or Traffic or Vehicle emissions or Vaping or Water)).ti,ab,kf.	3010

11	limit 10 to yr="2014 -Current"	1139
12	limit 11 to "review articles"	153
13	8 and 11	92
14	7 or 9 or 12 or 13 [REVIEWS]	400
15	6 not 14 [results with Reviews excluded]	1668

Scopus (Elsevier)

3	((TITLE-ABS-KEY ((adenoma* OR astrocytoma* OR blastoma* OR cancer* OR carcinoma* OR chondrosarcoma* OR chordoma* OR craniopharyngioma* OR ependymoma* OR fibrosarcoma* OR gangliocytoma* OR ganglioglioma* OR ganglioma* OR ganglioneuroblastoma* OR germinoma* OR glioblastoma* OR glioma* OR hemangioblastoma* OR hepatoblastoma* OR ilcrs OR leuk*emia* OR lymphoma* OR malignan* OR medulloblastoma* OR melanoma* OR meningioma* OR "Myelodysplastic syndrome*" OR "Myeloproliferative disease*" OR neoplasm* OR nephroblastoma* OR neuroblastoma OR neuroma* OR oligodendroglioma* OR oncology OR osteosarcoma* OR pineoblastoma* OR pineocytoma* OR retinoblastoma* OR rhabdomyosarcoma* OR rms OR sarcoma* OR schwannoma* OR tumor* OR tumour*) W/3 (adolescen* OR child* OR infan* OR neonat* OR p*ediatric* OR prenatal))) AND (TITLE (risk* OR etiolog* OR aetiolog* OR caus* OR cluster* OR epidemiol* OR protect*) OR KEY (risk* OR etiolog* OR aetiolog* OR caus* OR cluster* OR epidemiol* OR protect*) OR ABS (risk* W/7 (adenoma* OR astrocytoma* OR blastoma* OR cancer* OR carcinoma* OR "Childhood all" OR chondrosarcoma* OR chordoma* OR craniopharyngioma* OR ependymoma* OR fibrosarcoma* OR gangliocytoma* OR ganglioglioma* OR ganglioma* OR ganglioneuroblastoma* OR germinoma* OR glioblastoma* OR glioma* OR hemangioblastoma* OR hepatoblastoma* OR ilcrs OR leuk*emia* OR lymphoma* OR malignan* OR medulloblastoma* OR melanoma* OR meningioma* OR "Myelodysplastic syndrome*" OR "Myeloproliferative disease*" OR neoplasm* OR nephroblastoma* OR neuroblastoma OR neuroma* OR oligodendroglioma* OR oncology OR osteosarcoma* OR pineoblastoma* OR pineocytoma* OR retinoblastoma* OR rhabdomyosarcoma* OR rms OR sarcoma* OR schwannoma* OR tumor* OR tumour*))) AND (TITLE-ABS-KEY (agrochemical* OR arsenic OR "Assisted reproductive technolog*" OR "Birth defect*" OR "Birth order*" OR birthweight OR "Birth weight" OR "Bisphenol A" OR bpa OR breastfeed* OR "Breast-feed*" OR "Breast milk" OR carcinogen* OR chemical* OR circadian OR cluster* OR contamin* OR "Day care" OR daycare OR des OR diethylstilbestrol OR diethylstilboestrol OR diesel OR "Diagnostic x-ray" OR dioxin OR dye* OR ecigarette* OR "e-cigarette*" OR "Electric transmission" OR "Electromagnetic fields" OR "Endocrine disruptor*" OR environment* OR epigenetic* OR exposure* OR "Fertility treatment*" OR "Flame retardant*" OR fluorocarbons OR food* OR gasoline OR "Gene-environment*" OR "Hazardous waste" OR "Heavier at birth" OR "Human milk" OR hydrocarbon* OR "In vitro fertilization" OR infection* OR insecticide* OR irradiation OR ivf OR maternal OR "Medical imaging" OR "Nuclear plant*" OR "Nuclear reactor*" OR obesity OR parental OR paternal OR "Perfluorooctanoic acid" OR pfoa OR pesticide* OR petrochemical OR petroleum OR "Perfluorooctanesulfo	2,917 document results

	nic<br>acid" OR pfas OR pfos OR phenol* OR phototherapy OR plastic* OR pollut* OR "Polyc yclic Aromatic Hydrocarbons" OR preeclampsia OR "Pre-<br>eclampsia" OR preterm OR radiation OR radon OR "Season of<br>birth" OR smok* OR soil OR solvent* OR sunscreen OR traffic OR "Vehicle<br>emissions" OR vaping OR water)) AND (TITLE-ABS-KEY<br>(associated OR association* OR "Attributable risk*" OR "Case control" OR "Odds<br>ratio*" OR "Rate ratio*" OR "Relative risk" OR regression OR "Risk<br>ratio")) AND PUBYEAR > 2013) AND NOT ((((TITLE-ABS<br>(((adenoma* OR astrocytoma* OR blastoma* OR cancer* OR carcinoma* OR chondros arcoma* OR chordoma* OR craniopharyngioma* OR ependymoma* OR fibrosarcoma* OR gangliocytoma* OR ganglioglioma* OR ganglioma* OR ganglioneuroblastoma* OR ge rminoma* OR glioblastoma* OR glioma* OR hemangioblastoma* OR hepatoblastoma* OR ilcrs OR leuk*emia* OR lymphoma* OR malignan* OR medulloblastoma* OR melan oma* OR meningioma* OR "Myelodysplastic syndrome*" OR "Myeloproliferative<br>disease*" OR neoplasm* OR nephroblastoma* OR neuroblastoma OR neuroma* OR olig odendroglioma* OR oncology OR osteosarcoma* OR pineoblastoma* OR pineocytoma* OR retinoblastoma* OR rhabdomyosarcoma* OR rms OR sarcoma* OR schwannoma* O R tumor* OR tumour*) W/3 (adolescen* OR child* OR infan* OR neonat* OR p*ediat ric* OR prenatal)) W/9 (risk* OR etiolog* OR aetiolog* OR caus* OR cluster* OR epi demiol* OR protect*) W/9 (agrochemical* OR arsenic OR "Assisted reproductive<br>technolog*" OR "Birth defect*" OR "Birth order*" OR birthweight OR "Birth<br>weight" OR "Bisphenol A" OR bpa OR breastfeed* OR "Breast-feed*" OR "Breast<br>milk" OR carcinogen* OR chemical* OR circadian OR cluster* OR contamin* OR "Day<br>care" OR daycare OR des OR diethylstilbestrol OR diethylstilboestrol OR diesel OR "Dia gnostic x-ray" OR dioxin OR dye* OR ecigarette* OR "e-cigarette*" OR "Electric<br>transmission" OR "Electromagnetic fields" OR "Endocrine<br>disruptor*" OR environment* OR epigenetic* OR exposure* OR "Fertility<br>treatment*" OR "Flame retardant*" OR fluorocarbons OR food* OR gasoline OR "Gene-<br>environment*" OR "Hazardous waste" OR "Heavier at birth" OR "Human<br>milk" OR hydrocarbon* OR "In vitro<br>fertilization" OR infection* OR insecticide* OR irradiation OR ivf OR maternal OR "Medi cal imaging" OR "Nuclear plant*" OR "Nuclear<br>reactor*" OR obesity OR parental OR paternal OR "Perfluorooctanoic<br>acid" OR pfoa OR pesticide* OR petrochemical OR petroleum OR "Perfluorooctanesulfo nic<br>acid" OR pfas OR pfos OR phenol* OR phototherapy OR plastic* OR pollut* OR "Polyc yclic Aromatic Hydrocarbons" OR preeclampsia OR "Pre-<br>eclampsia" OR preterm OR radiation OR radon OR "Season of<br>birth" OR smok* OR soil OR solvent* OR sunscreen OR traffic OR "Vehicle<br>emissions" OR vaping OR water)) AND PUBYEAR > 2013) AND (DOCTYPE<br>(re))) OR ((TITLE-ABS<br>(((adenoma* OR astrocytoma* OR blastoma* OR cancer* OR carcinoma* OR chondros arcoma* OR chordoma* OR craniopharyngioma* OR ependymoma* OR fibrosarcoma* OR gangliocytoma* OR ganglioglioma* OR ganglioma* OR ganglioneuroblastoma* OR ge rminoma* OR glioblastoma* OR glioma* OR hemangioblastoma* OR hepatoblastoma* OR ilcrs OR leuk*emia* OR lymphoma* OR malignan* OR medulloblastoma* OR melan oma* OR meningioma* OR "Myelodysplastic syndrome*" OR "Myeloproliferative<br>disease*" OR neoplasm* OR nephroblastoma* OR neuroblastoma OR neuroma* OR olig odendroglioma* OR oncology OR osteosarcoma* OR pineoblastoma* OR pineocytoma* OR retinoblastoma* OR rhabdomyosarcoma* OR rms OR sarcoma* OR schwannoma* O R tumor* OR tumour*) W/3 (adolescen* OR child* OR infan* OR neonat* OR p*ediat	

	ric* OR prenatal)) W/9 (risk* OR etiolog* OR aetiolog* OR caus* OR cluster* OR epi demiol* OR protect*) W/9 (agrochemical* OR arsenic OR "Assisted reproductive technolog*" OR "Birth defect*" OR "Birth order*" OR birthweight OR "Birth weight" OR "Bisphenol A" OR bpa OR breastfeed* OR "Breast-feed*" OR "Breast milk" OR carcinogen* OR chemical* OR circadian OR cluster* OR contamin* OR "Day care" OR daycare OR des OR diethylstilbestrol OR diethylstilboestrol OR diesel OR "Dia gnostic x-ray" OR dioxin OR dye* OR ecigarette* OR "e-cigarette*" OR "Electric transmission" OR "Electromagnetic fields" OR "Endocrine disruptor*" OR environment* OR epigenetic* OR exposure* OR "Fertility treatment*" OR "Flame retardant*" OR fluorocarbons OR food* OR gasoline OR "Gene-environment*" OR "Hazardous waste" OR "Heavier at birth" OR "Human milk" OR hydrocarbon* OR "In vitro fertilization" OR infection* OR insecticide* OR irradiation OR ivf OR maternal OR "Medi cal imaging" OR "Nuclear plant*" OR "Nuclear reactor*" OR obesity OR parental OR paternal OR "Perfluorooctanoic acid" OR pfoa OR pesticide* OR petrochemical OR petroleum OR "Perfluorooctanesulfo nic acid" OR pfas OR pfos OR phenol* OR phototherapy OR plastic* OR pollut* OR "Polyc yclic Aromatic Hydrocarbons" OR preeclampsia OR "Pre-eclampsia" OR preterm OR radiation OR radon OR "Season of birth" OR smok* OR soil OR solvent* OR sunscreen OR traffic OR "Vehicle emissions" OR vaping OR water)) AND PUBYEAR > 2013) AND (TITLE (metaanalysis OR meta-analysis OR review OR scoping OR systematic)))) OR ((((TITLE-ABS-KEY ((adenoma* OR astrocytoma* OR blastoma* OR cancer* OR carcinoma* OR chondrosa rcoma* OR chordoma* OR craniopharyngioma* OR ependymoma* OR fibrosarcoma* O R gangliocytoma* OR ganglioglioma* OR ganglioma* OR ganglioneuroblastoma* OR ger minoma* OR glioblastoma* OR glioma* OR hemangioblastoma* OR hepatoblastoma* O R ilcrs OR leuk*emia* OR lymphoma* OR malignan* OR medulloblastoma* OR melano ma* OR meningioma* OR "Myelodysplastic syndrome*" OR "Myeloproliferative disease*" OR neoplasm* OR nephroblastoma* OR neuroblastoma OR neuroma* OR olig odendroglioma* OR oncology OR osteosarcoma* OR pineoblastoma* OR pineocytoma* OR retinoblastoma* OR rhabdomyosarcoma* OR rms OR sarcoma* OR schwannoma* O R tumor* OR tumour*) W/3 (adolescen* OR child* OR infan* OR neonat* OR p*ediat ric* OR prenatal))) AND (TITLE (risk* OR etiolog* OR aetiolog* OR caus* OR cluster* OR epidemiol* OR protect*) OR KEY (risk* OR etiolog* OR aetiolog* OR caus* OR cluster* OR epidemiol* OR protect*) OR ABS (risk* W/7 (adenoma* OR astrocytoma* OR blastoma* OR cancer* OR carcinoma* OR "Childhood all" OR chondrosarcoma* OR chordoma* OR craniopharyngioma* OR ependymoma* OR fibrosarcoma* OR gangliocytoma* OR ganglioglioma* OR ganglioma* OR ganglioneurob lastoma* OR germinoma* OR glioblastoma* OR glioma* OR hemangioblastoma* OR he patoblastoma* OR ilcrs OR leuk*emia* OR lymphoma* OR malignan* OR medulloblasto ma* OR melanoma* OR meningioma* OR "Myelodysplastic syndrome*" OR "Myeloproliferative disease*" OR neoplasm* OR nephroblastoma* OR neuroblastoma OR neuroma* OR olig odendroglioma* OR oncology OR osteosarcoma* OR pineoblastoma* OR pineocytoma* OR retinoblastoma* OR rhabdomyosarcoma* OR rms OR sarcoma* OR schwannoma* O R tumor* OR tumour*))) AND (TITLE-ABS-KEY (agrochemical* OR arsenic OR "Assisted reproductive technolog*" OR "Birth defect*" OR "Birth order*" OR birthweight OR "Birth	

	weight" OR "Bisphenol A" OR bpa OR breastfeed* OR "Breast-feed*" OR "Breast milk" OR carcinogen* OR chemical* OR circadian OR cluster* OR contamin* OR "Day care" OR daycare OR des OR diethylstilbestrol OR diethylstilboestrol OR diesel OR "Dia gnostic x-ray" OR dioxin OR dye* OR ecigarette* OR "e-cigarette*" OR "Electric transmission" OR "Electromagnetic fields" OR "Endocrine disruptor*" OR environment* OR epigenetic* OR exposure* OR "Fertility treatment*" OR "Flame retardant*" OR fluorocarbons OR food* OR gasoline OR "Gene-environment*" OR "Hazardous waste" OR "Heavier at birth" OR "Human milk" OR hydrocarbon* OR "In vitro fertilization" OR infection* OR insecticide* OR irradiation OR ivf OR maternal OR "Medi cal imaging" OR "Nuclear plant*" OR "Nuclear reactor*" OR obesity OR parental OR paternal OR "Perfluorooctanoic acid" OR pfoa OR pesticide* OR petrochemical OR petroleum OR "Perfluorooctanesulfo nic acid" OR pfas OR pfos OR phenol* OR phototherapy OR plastic* OR pollut* OR "Polyc yclic Aromatic Hydrocarbons" OR preeclampsia OR "Pre-eclampsia" OR preterm OR radiation OR radon OR "Season of birth" OR smok* OR soil OR solvent* OR sunscreen OR traffic OR "Vehicle emissions" OR vaping OR water)) AND (TITLE-ABS-KEY (associated OR association* OR "Attributable risk*" OR "Case control" OR "Odds ratio*" OR "Rate ratio*" OR "Relative risk" OR regression OR "Risk ratio")) AND PUBYEAR > 2013) AND (DOCTYPE (re))) OR (((TITLE-ABS-KEY ((adenoma* OR astrocytoma* OR blastoma* OR cancer* OR carcinoma* OR chondrosa rcoma* OR chordoma* OR craniopharyngioma* OR ependymoma* OR fibrosarcoma* O R gangliocytoma* OR ganglioglioma* OR ganglioma* OR ganglioneuroblastoma* OR ger minoma* OR glioblastoma* OR glioma* OR hemangioblastoma* OR hepatoblastoma* O R ilcrs OR leuk*emia* OR lymphoma* OR malignan* OR medulloblastoma* OR melano ma* OR meningioma* OR "Myelodysplastic syndrome*" OR "Myeloproliferative disease*" OR neoplasm* OR nephroblastoma* OR neuroblastoma OR neuroma* OR olig odendroglioma* OR oncology OR osteosarcoma* OR pineoblastoma* OR pineocytoma* OR retinoblastoma* OR rhabdomyosarcoma* OR rms OR sarcoma* OR schwannoma* O R tumor* OR tumour*) W/3 (adolescen* OR child* OR infan* OR neonat* OR p*ediat ric* OR prenatal))) AND (TITLE (risk* OR etiolog* OR aetiolog* OR caus* OR cluster* OR epidemiol* OR protect*) OR KEY (risk* OR etiolog* OR aetiolog* OR caus* OR cluster* OR epidemiol* OR protect*) OR ABS (risk* W/7 (adenoma* OR astrocytoma* OR blastoma* OR cancer* OR carcinoma* OR "Childhood all" OR chondrosarcoma* OR chordoma* OR craniopharyngioma* OR ependymoma* OR fibrosarcoma* OR gangliocytoma* OR ganglioglioma* OR ganglioma* OR ganglioneurob lastoma* OR germinoma* OR glioblastoma* OR glioma* OR hemangioblastoma* OR he patoblastoma* OR ilcrs OR leuk*emia* OR lymphoma* OR malignan* OR medulloblasto ma* OR melanoma* OR meningioma* OR "Myelodysplastic syndrome*" OR "Myeloproliferative disease*" OR neoplasm* OR nephroblastoma* OR neuroblastoma OR neuroma* OR olig odendroglioma* OR oncology OR osteosarcoma* OR pineoblastoma* OR pineocytoma* OR retinoblastoma* OR rhabdomyosarcoma* OR rms OR sarcoma* OR schwannoma* O R tumor* OR tumour*))) AND (TITLE-ABS-KEY (agrochemical* OR arsenic OR "Assisted reproductive technolog*" OR "Birth defect*" OR "Birth order*" OR birthweight OR "Birth weight" OR "Bisphenol A" OR bpa OR breastfeed* OR "Breast-feed*" OR "Breast milk" OR carcinogen* OR chemical* OR circadian OR cluster* OR contamin* OR "Day	

	care" OR daycare OR des OR diethylstilbestrol OR diethylstilboestrol OR diesel OR "Dia gnostic x-ray" OR dioxin OR dye* OR ecigarette* OR "e-cigarette*" OR "Electric transmission" OR "Electromagnetic fields" OR "Endocrine disruptor*" OR environment* OR epigenetic* OR exposure* OR "Fertility treatment*" OR "Flame retardant*" OR fluorocarbons OR food* OR gasoline OR "Gene-environment*" OR "Hazardous waste" OR "Heavier at birth" OR "Human milk" OR hydrocarbon* OR "In vitro fertilization" OR infection* OR insecticide* OR irradiation OR ivf OR maternal OR "Medical imaging" OR "Nuclear plant*" OR"Nuclear reactor*" OR obesity OR parental OR paternal OR "Perfluorooctanoic acid" OR pfoa OR pesticide* OR petrochemical OR petroleum OR "Perfluorooctanesulfo nic acid" OR pfas OR pfos OR phenol* OR phototherapy OR plastic* OR pollut* OR "Polycyclic Aromatic Hydrocarbons" OR preeclampsia OR "Pre-eclampsia" OR preterm OR radiation OR radon OR "Season of birth" OR smok* OR soil OR solvent* OR sunscreen OR traffic OR "Vehicle emissions" OR vaping OR water)) AND (TITLE-ABS-KEY (associated OR association* OR "Attributable risk*" OR "Case control" OR "Odds ratio*" OR "Rate ratio*" OR "Relative risk" OR regression OR "Risk ratio")) AND PUBYEAR > 2013) AND (TITLE (metaanalysis OR meta-analysis OR review OR scoping OR systematic)))))	
2	(((TITLE-ABS (((adenoma* OR astrocytoma* OR blastoma* OR cancer* OR carcinoma* OR chondros arcoma* OR chordoma* OR craniopharyngioma* OR ependymoma* OR fibrosarcoma* OR gangliocytoma* OR ganglioglioma* OR ganglioma* OR ganglioneuroblastoma* OR ge rminoma* OR glioblastoma* OR glioma* OR hemangioblastoma* OR hepatoblastoma* OR ilcrs OR leuk*emia* OR lymphoma* OR malignan* OR medulloblastoma* OR melan oma* OR meningioma* OR "Myelodysplastic syndrome*" OR "Myeloproliferative disease*" OR neoplasm* OR nephroblastoma* OR neuroblastoma OR neuroma* OR olig odendroglioma* OR oncology OR osteosarcoma* OR pineoblastoma* OR pineocytoma* OR retinoblastoma* OR rhabdomyosarcoma* OR rms OR sarcoma* OR schwannoma* O R tumor* OR tumour*) W/3 (adolescen* OR child* OR infan* OR neonat* OR p*ediat ric* OR prenatal)) W/9 (risk* OR etiolog* OR aetiolog* OR caus* OR cluster* OR epi demiol* OR protect*) W/9 (agrochemical* OR arsenic OR "Assisted reproductive technolog*" OR "Birth defect*" OR "Birth order*" OR birthweight OR "Birth weight" OR "Bisphenol A" OR bpa OR breastfeed* OR "Breast-feed*" OR "Breast milk" OR carcinogen* OR chemical* OR circadian OR cluster* OR contamin* OR "Day care" OR daycare OR des OR diethylstilbestrol OR diethylstilboestrol OR diesel OR "Dia gnostic x-ray" OR dioxin OR dye* OR ecigarette* OR "e-cigarette*" OR "Electric transmission" OR "Electromagnetic fields" OR "Endocrine disruptor*" OR environment* OR epigenetic* OR exposure* OR "Fertility treatment*" OR "Flame retardant*" OR fluorocarbons OR food* OR gasoline OR "Gene-environment*" OR "Hazardous waste" OR "Heavier at birth" OR "Human milk" OR hydrocarbon* OR "In vitro fertilization" OR infection* OR insecticide* OR irradiation OR ivf OR maternal OR "Medi cal imaging" OR "Nuclear plant*" OR "Nuclear reactor*" OR obesity OR parental OR paternal OR "Perfluorooctanoic acid" OR pfoa OR pesticide* OR petrochemical OR petroleum OR "Perfluorooctanesulfo nic acid" OR pfas OR pfos OR phenol* OR phototherapy OR plastic* OR pollut* OR "Polyc yclic Aromatic Hydrocarbons" OR preeclampsia OR "Pre-eclampsia" OR preterm OR radiation OR radon OR "Season of	667 document results

	birth" OR smok* OR soil OR solvent* OR sunscreen OR traffic OR "Vehicle<br>emissions" OR vaping OR water)) AND PUBYEAR > 2013) AND (DOCTYPE<br>(re))) OR ((TITLE-ABS<br>(((adenoma* OR astrocytoma* OR blastoma* OR cancer* OR carcinoma* OR chondros<br>arcoma* OR chordoma* OR craniopharyngioma* OR ependymoma* OR fibrosarcoma*<br>OR gangliocytoma* OR ganglioglioma* OR ganglioma* OR ganglioneuroblastoma* OR ge<br>rminoma* OR glioblastoma* OR glioma* OR hemangioblastoma* OR hepatoblastoma*<br>OR ilcrs OR leuk*emia* OR lymphoma* OR malignan* OR medulloblastoma* OR melan<br>oma* OR meningioma* OR "Myelodysplastic syndrome*" OR "Myeloproliferative<br>disease*" OR neoplasm* OR nephroblastoma* OR neuroblastoma OR neuroma* OR olig<br>odendroglioma* OR oncology OR osteosarcoma* OR pineoblastoma* OR pineocytoma*<br>OR retinoblastoma* OR rhabdomyosarcoma* OR rms OR sarcoma* OR schwannoma* O<br>R tumor* OR tumour*) W/3 (adolescen* OR child* OR infan* OR neonat* OR p*ediat<br>ric* OR prenatal)) W/9 (risk* OR etiolog* OR aetiolog* OR caus* OR cluster* OR epi<br>demiol* OR protect*) W/9 (agrochemical* OR arsenic OR "Assisted reproductive<br>technolog*" OR "Birth defect*" OR "Birth order*" OR birthweight OR "Birth<br>weight" OR "Bisphenol A" OR bpa OR breastfeed* OR "Breast-feed*" OR "Breast<br>milk" OR carcinogen* OR chemical* OR circadian OR cluster* OR contamin* OR "Day<br>care" OR daycare OR des OR diethylstilbestrol OR diethylstilboestrol OR diesel OR "Dia<br>gnostic x-ray" OR dioxin OR dye* OR ecigarette* OR "e-cigarette*" OR "Electric<br>transmission" OR "Electromagnetic fields" OR "Endocrine<br>disruptor*" OR environment* OR epigenetic* OR exposure* OR "Fertility<br>treatment*" OR "Flame retardant*" OR fluorocarbons OR food* OR gasoline OR "Gene-<br>environment*" OR "Hazardous waste" OR "Heavier at birth" OR "Human<br>milk" OR hydrocarbon* OR "In vitro<br>fertilization" OR infection* OR insecticide* OR irradiation OR ivf OR maternal OR "Medi<br>cal imaging" OR "Nuclear plant*" OR "Nuclear<br>reactor*" OR obesity OR parental OR paternal OR "Perfluorooctanoic<br>acid" OR pfoa OR pesticide* OR petrochemical OR petroleum OR "Perfluorooctanesulfo<br>nic<br>acid" OR pfas OR pfos OR phenol* OR phototherapy OR plastic* OR pollut* OR "Polyc<br>yclic Aromatic Hydrocarbons" OR preeclampsia OR "Pre-<br>eclampsia" OR preterm OR radiation OR radon OR "Season of<br>birth" OR smok* OR soil OR solvent* OR sunscreen OR traffic OR "Vehicle<br>emissions" OR vaping OR water)) AND PUBYEAR > 2013) AND (TITLE<br>(metaanalysis OR meta-<br>analysis OR review OR scoping OR systematic)))) OR ((((TITLE-ABS-KEY<br>((adenoma* OR astrocytoma* OR blastoma* OR cancer* OR carcinoma* OR chondrosa<br>rcoma* OR chordoma* OR craniopharyngioma* OR ependymoma* OR fibrosarcoma* O<br>R gangliocytoma* OR ganglioglioma* OR ganglioma* OR ganglioneuroblastoma* OR ger<br>minoma* OR glioblastoma* OR glioma* OR hemangioblastoma* OR hepatoblastoma* O<br>R ilcrs OR leuk*emia* OR lymphoma* OR malignan* OR medulloblastoma* OR melano<br>ma* OR meningioma* OR "Myelodysplastic syndrome*" OR "Myeloproliferative<br>disease*" OR neoplasm* OR nephroblastoma* OR neuroblastoma OR neuroma* OR olig<br>odendroglioma* OR oncology OR osteosarcoma* OR pineoblastoma* OR pineocytoma*<br>OR retinoblastoma* OR rhabdomyosarcoma* OR rms OR sarcoma* OR schwannoma* O<br>R tumor* OR tumour*) W/3 (adolescen* OR child* OR infan* OR neonat* OR p*ediat<br>ric* OR prenatal))) AND (TITLE<br>(risk* OR etiolog* OR aetiolog* OR caus* OR cluster* OR epidemiol* OR protect*) OR<br>KEY<br>(risk* OR etiolog* OR aetiolog* OR caus* OR cluster* OR epidemiol* OR protect*) OR<br>ABS	

	(risk* W/7 (adenoma* OR astrocytoma* OR blastoma* OR cancer* OR carcinoma* OR "Childhood<br>all" OR chondrosarcoma* OR chordoma* OR craniopharyngioma* OR ependymoma* OR<br>fibrosarcoma* OR gangliocytoma* OR ganglioglioma* OR ganglioma* OR ganglioneurob<br>lastoma* OR germinoma* OR glioblastoma* OR glioma* OR hemangioblastoma* OR he<br>patoblastoma* OR ilcrs OR leuk*emia* OR lymphoma* OR malignan* OR medulloblasto<br>ma* OR melanoma* OR meningioma* OR "Myelodysplastic<br>syndrome*" OR "Myeloproliferative<br>disease*" OR neoplasm* OR nephroblastoma* OR neuroblastoma OR neuroma* OR olig<br>odendroglioma* OR oncology OR osteosarcoma* OR pineoblastoma* OR pineocytoma*<br>OR retinoblastoma* OR rhabdomyosarcoma* OR rms OR sarcoma* OR schwannoma* O<br>R tumor* OR tumour*))) AND (TITLE-ABS-KEY (agrochemical* OR arsenic OR "Assisted<br>reproductive technolog*" OR "Birth defect*" OR "Birth order*" OR birthweight OR "Birth<br>weight" OR "Bisphenol A" OR bpa OR breastfeed* OR "Breast-feed*" OR "Breast<br>milk" OR carcinogen* OR chemical* OR circadian OR cluster* OR contamin* OR "Day<br>care" OR daycare OR des OR diethylstilbestrol OR diethylstilboestrol OR diesel OR "Dia<br>gnostic x-ray" OR dioxin OR dye* OR ecigarette* OR "e-cigarette*" OR "Electric<br>transmission" OR "Electromagnetic fields" OR "Endocrine<br>disruptor*" OR environment* OR epigenetic* OR exposure* OR "Fertility<br>treatment*" OR "Flame retardant*" OR fluorocarbons OR food* OR gasoline OR "Gene-<br>environment*" OR "Hazardous waste" OR "Heavier at birth" OR "Human<br>milk" OR hydrocarbon* OR "In vitro<br>fertilization" OR infection* OR insecticide* OR irradiation OR ivf OR maternal OR "Medi<br>cal imaging" OR "Nuclear plant*" OR "Nuclear<br>reactor*" OR obesity OR parental OR paternal OR "Perfluorooctanoic<br>acid" OR pfoa OR pesticide* OR petrochemical OR petroleum OR "Perfluorooctanesulfo<br>nic<br>acid" OR pfas OR pfos OR phenol* OR phototherapy OR plastic* OR pollut* OR "Polyc<br>yclic Aromatic Hydrocarbons" OR preeclampsia OR "Pre-<br>eclampsia" OR preterm OR radiation OR radon OR "Season of<br>birth" OR smok* OR soil OR solvent* OR sunscreen OR traffic OR "Vehicle<br>emissions" OR vaping OR water)) AND (TITLE-ABS-KEY<br>(associated OR association* OR "Attributable risk*" OR "Case control" OR "Odds<br>ratio*" OR "Rate ratio*" OR "Relative risk" OR regression OR "Risk<br>ratio")) AND PUBYEAR > 2013) AND (DOCTYPE (re))) OR (((TITLE-ABS-KEY<br>((adenoma* OR astrocytoma* OR blastoma* OR cancer* OR carcinoma* OR chondrosa<br>rcoma* OR chordoma* OR craniopharyngioma* OR ependymoma* OR fibrosarcoma* O<br>R gangliocytoma* OR ganglioglioma* OR ganglioma* OR ganglioneuroblastoma* OR ger<br>minoma* OR glioblastoma* OR glioma* OR hemangioblastoma* OR hepatoblastoma* O<br>R ilcrs OR leuk*emia* OR lymphoma* OR malignan* OR medulloblastoma* OR melano<br>ma* OR meningioma* OR "Myelodysplastic syndrome*" OR "Myeloproliferative<br>disease*" OR neoplasm* OR nephroblastoma* OR neuroblastoma OR neuroma* OR olig<br>odendroglioma* OR oncology OR osteosarcoma* OR pineoblastoma* OR pineocytoma*<br>OR retinoblastoma* OR rhabdomyosarcoma* OR rms OR sarcoma* OR schwannoma* O<br>R tumor* OR tumour*) W/3 (adolescen* OR child* OR infan* OR neonat* OR p*ediat<br>ric* OR prenatal))) AND (TITLE<br>(risk* OR etiolog* OR aetiolog* OR caus* OR cluster* OR epidemiol* OR protect*) OR<br>KEY<br>(risk* OR etiolog* OR aetiolog* OR caus* OR cluster* OR epidemiol* OR protect*) OR<br>ABS<br>(risk* W/7 (adenoma* OR astrocytoma* OR blastoma* OR cancer* OR carcinoma* OR "Childhood	

	all" OR chondrosarcoma* OR chordoma* OR craniopharyngioma* OR ependymoma* OR fibrosarcoma* OR gangliocytoma* OR ganglioglioma* OR ganglioma* OR ganglioneurob lastoma* OR germinoma* OR glioblastoma* OR glioma* OR hemangioblastoma* OR he patoblastoma* OR ilcrs OR leuk*emia* OR lymphoma* OR malignan* OR medulloblasto ma* OR melanoma* OR meningioma* OR "Myelodysplastic syndrome*" OR "Myeloproliferative disease*" OR neoplasm* OR nephroblastoma* OR neuroblastoma OR neuroma* OR olig odendroglioma* OR oncology OR osteosarcoma* OR pineoblastoma* OR pineocytoma* OR retinoblastoma* OR rhabdomyosarcoma* OR rms OR sarcoma* OR schwannoma* O R tumor* OR tumour*))) AND (TITLE-ABS-KEY (agrochemical* OR arsenic OR "Assisted reproductive technolog*" OR "Birth defect*" OR "Birth order*" OR birthweight OR "Birth weight" OR "Bisphenol A" OR bpa OR breastfeed* OR "Breast-feed*" OR "Breast milk" OR carcinogen* OR chemical* OR circadian OR cluster* OR contamin* OR "Day care" OR daycare OR des OR diethylstilbestrol OR diethylstilboestrol OR diesel OR "Dia gnostic x-ray" OR dioxin OR dye* OR ecigarette* OR "e-cigarette*" OR "Electric transmission" OR "Electromagnetic fields" OR "Endocrine disruptor*" OR environment* OR epigenetic* OR exposure* OR "Fertility treatment*" OR "Flame retardant*" OR fluorocarbons OR food* OR gasoline OR "Gene-environment*" OR "Hazardous waste" OR "Heavier at birth" OR "Human milk" OR hydrocarbon* OR "In vitro fertilization" OR infection* OR insecticide* OR irradiation OR ivf OR maternal OR "Medi cal imaging" OR "Nuclear plant*" OR "Nuclear reactor*" OR obesity OR parental OR paternal OR "Perfluorooctanoic acid" OR pfoa OR pesticide* OR petrochemical OR petroleum OR "Perfluorooctanesulfo nic acid" OR pfas OR pfos OR phenol* OR phototherapy OR plastic* OR pollut* OR "Polyc yclic Aromatic Hydrocarbons" OR preeclampsia OR "Pre-eclampsia" OR preterm OR radiation OR radon OR "Season of birth" OR smok* OR soil OR solvent* OR sunscreen OR traffic OR "Vehicle emissions" OR vaping OR water)) AND (TITLE-ABS-KEY (associated OR association* OR "Attributable risk*" OR "Case control" OR "Odds ratio*" OR "Rate ratio*" OR "Relative risk" OR regression OR "Risk ratio")) AND PUBYEAR > 2013) AND (TITLE (metaanalysis OR meta-analysis OR review OR scoping OR systematic))))	
1	(TITLE-ABS-KEY ((adenoma* OR astrocytoma* OR blastoma* OR cancer* OR carcinoma* OR chondrosa rcoma* OR chordoma* OR craniopharyngioma* OR ependymoma* OR fibrosarcoma* O R gangliocytoma* OR ganglioglioma* OR ganglioma* OR ganglioneuroblastoma* OR ger minoma* OR glioblastoma* OR glioma* OR hemangioblastoma* OR hepatoblastoma* O R ilcrs OR leuk*emia* OR lymphoma* OR malignan* OR medulloblastoma* OR melano ma* OR meningioma* OR "Myelodysplastic syndrome*" OR "Myeloproliferative disease*" OR neoplasm* OR nephroblastoma* OR neuroblastoma OR neuroma* OR olig odendroglioma* OR oncology OR osteosarcoma* OR pineoblastoma* OR pineocytoma* OR retinoblastoma* OR rhabdomyosarcoma* OR rms OR sarcoma* OR schwannoma* O R tumor* OR tumour*) W/3 (adolescen* OR child* OR infan* OR neonat* OR p*ediat ric* OR prenatal))) AND (TITLE (risk* OR etiolog* OR aetiolog* OR caus* OR cluster* OR epidemiol* OR protect*) OR KEY (risk* OR etiolog* OR aetiolog* OR caus* OR cluster* OR epidemiol* OR protect*) OR ABS (risk* W/7 (adenoma* OR astrocytoma* OR blastoma* OR cancer* OR carcinoma* OR	3,474 document results

	"Childhood all" OR chondrosarcoma* OR chordoma* OR craniopharyngioma* OR ependymoma* OR fibrosarcoma* OR gangliocytoma* OR ganglioglioma* OR ganglioma* OR ganglioneuroblastoma* OR germinoma* OR glioblastoma* OR glioma* OR hemangioblastoma* OR hepatoblastoma* OR ilcrs OR leuk*emia* OR lymphoma* OR malignan* OR medulloblastoma* OR melanoma* OR meningioma* OR "Myelodysplastic syndrome*" OR "Myeloproliferative disease*" OR neoplasm* OR nephroblastoma* OR neuroblastoma OR neuroma* OR oligodendroglioma* OR oncology OR osteosarcoma* OR pineoblastoma* OR pineocytoma* OR retinoblastoma* OR rhabdomyosarcoma* OR rms OR sarcoma* OR schwannoma* OR tumor* OR tumour*))) AND (TITLE-ABS-KEY (agrochemical* OR arsenic OR "Assisted reproductive technolog*" OR "Birth defect*" OR "Birth order*" OR birthweight OR "Birth weight" OR "Bisphenol A" OR bpa OR breastfeed* OR "Breast-feed*" OR "Breast milk" OR carcinogen* OR chemical* OR circadian OR cluster* OR contamin* OR "Day care" OR daycare OR des OR diethylstilbestrol OR diethylstilboestrol OR diesel OR "Diagnostic x-ray" OR dioxin OR dye* OR ecigarette* OR "e-cigarette*" OR "Electric transmission" OR "Electromagnetic fields" OR "Endocrine disruptor*" OR environment* OR epigenetic* OR exposure* OR "Fertility treatment*" OR "Flame retardant*" OR fluorocarbons OR food* OR gasoline OR "Gene-environment*" OR "Hazardous waste" OR "Heavier at birth" OR "Human milk" OR hydrocarbon* OR "In vitro fertilization" OR infection* OR insecticide* OR irradiation OR ivf OR maternal OR "Medical imaging" OR "Nuclear plant*" OR "Nuclear reactor*" OR obesity OR parental OR paternal OR "Perfluorooctanoic acid" OR pfoa OR pesticide* OR petrochemical OR petroleum OR "Perfluorooctanesulfonic acid" OR pfas OR pfos OR phenol* OR phototherapy OR plastic* OR pollut* OR "Polycyclic Aromatic Hydrocarbons" OR preeclampsia OR "Pre-eclampsia" OR preterm OR radiation OR radon OR "Season of birth" OR smok* OR soil OR solvent* OR sunscreen OR traffic OR "Vehicle emissions" OR vaping OR water)) AND (TITLE-ABS-KEY (associated OR association* OR "Attributable risk*" OR "Case control" OR "Odds ratio*" OR "Rate ratio*" OR "Relative risk" OR regression OR "Risk ratio")) AND PUBYEAR > 2013	
